# Regulation of CD4^+^ T Cell Signaling and Immunological Synapse by Protein Tyrosine Phosphatases: Molecular Mechanisms in Autoimmunity

**DOI:** 10.3389/fimmu.2019.01447

**Published:** 2019-06-26

**Authors:** Patricia Castro-Sánchez, Oscar Aguilar-Sopeña, Sergio Alegre-Gómez, Rocio Ramirez-Munoz, Pedro Roda-Navarro

**Affiliations:** ^1^Department of Immunology, Ophthalmology and ENT, School of Medicine, Complutense University, Madrid, Spain; ^2^Health Research Institute ‘12 de Octubre (imas12)', Madrid, Spain

**Keywords:** PTP, T cell activation, intracellular signaling, cytoskeleton, endosomal compartment, immunological synapse, autoimmunity

## Abstract

T cell activation and effector function is mediated by the formation of a long-lasting interaction established between T cells and antigen-presenting cells (APCs) called immunological synapse (IS). During T cell activation, different signaling molecules as well as the cytoskeleton and the endosomal compartment are polarized to the IS. This molecular dynamics is tightly regulated by phosphorylation networks, which are controlled by protein tyrosine phosphatases (PTPs). While some PTPs are known to be important regulators of adhesion, ligand discrimination or the stimulation threshold, there is still little information about the regulatory role of PTPs in cytoskeleton rearrangements and endosomal compartment dynamics. Besides, spatial and temporal regulation of PTPs and substrates at the IS is only barely known. Consistent with an important role of PTPs in T cell activation, multiple mutations as well as altered expression levels or dynamic behaviors have been associated with autoimmune diseases. However, the precise mechanism for the regulation of T cell activation and effector function by PTPs in health and autoimmunity is not fully understood. Herein, we review the current knowledge about the regulatory role of PTPs in CD4^+^ T cell activation, IS assembly and effector function. The potential molecular mechanisms mediating the action of these enzymes in autoimmune disorders are discussed.

## Introduction

**Figure 1:**
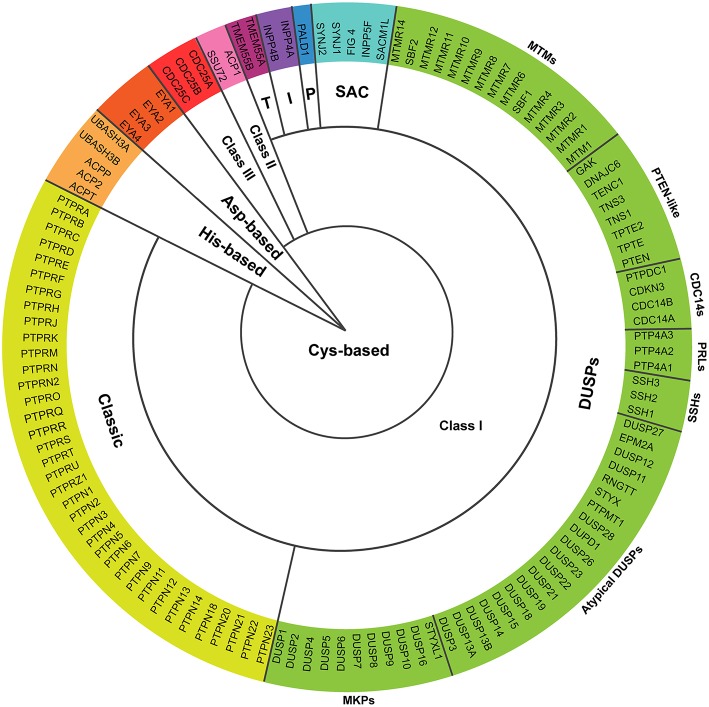
Proteins encoded by the extended PTPome. The schematic represents the 125 protein tyrosine phosphatases encoded by the extended PTPome and their classification following criteria by Alonso and Pulido (2). DUSPs, dual-specificity PTPs; MKPs, MAP kinase phosphatases; SSHs, slingshots; PRLs, phosphatases of regenerating liver; CDC14s, cell division cycle-14 proteins; PTEN, phosphatase and tensin homolog; MTMs, myotubularins; P, paladin; I, INPP4 phosphatases; T, TMEM55 phosphatases; CDC25, cell division cycle-25 protein.

Tight regulation of intracellular phosphorylation networks by kinase and phosphatase activities mediates cellular responses and prevents pathological disorders. In 2004, Alonso and co-workers postulated that 107 human genes code for protein tyrosine phosphatases (PTPs), characterized by conserved catalytic motifs and phosphatase domains (1). The superfamily of PTPs has recently been increased to 125 members, the so-called extended PTPome (2) (Figure 1 and Box 1). In addition to enzymes specific for phospho-Tyrosine (pTyr) residues, certain PTPs are able to dephosphorylate phospho-Serine (pSer) and phospho-Threonine (pThr) residues, phospholipids, or mRNA (1, 2).

Box 1 **Proteins encoded by the extended PTPome.**

**Cys-based**

***Class I***

The group of classical PTPs contains 37 enzymes specific for pTyr residues that includes receptor type PTPs (RPTP), which have a transmembrane domain, and non-receptor type PTPs (NRPTP), which are cytosolic or associated to different intracellular compartments.The group of dual-specificity PTPs (DUSPs) is the most diverse in terms of substrate specificity and contains 64 members including: (a) MAP kinase phosphatases (MKPs), which dephosphorylate both pTyr and pThr residues and present targeting motifs for MAPK; (b) atypical DUSPs, which lack specific MAPK targeting motifs and dephosphorylate either pTyr or pThr residues, with the exception of DUSP11, which dephosphorylates mRNA (3) and EPM2A, which dephosphorylates glycogen (4); (c) slingshots (SSHs; SSH1, SSH2, and SSH3), which activate cofilin by dephosphorylating the pSer-3 residue, and have actin-bundling activity (5); (d) phosphatases of regenerating liver (PRLs; PRL-1, PRL-2, and PRL-3), which have unknown substrates and have been implicated in cancer progression (6); (e) cell division cycle-14 proteins (CDC14s), which inactivate cyclin-dependent kinases (CDKs) to induce mitotic exit and are also involved in mitotic spindle formation (7, 8); (f) phosphatase and tensin homolog-like (PTEN-like) PTPs, which dephosphorylate the position 3 of phosphoinositides (9); and (g) myotubularins (MTMs) containing both, catalytically active members, which dephosphorylate the position 3 of PI3P and PI(3,5)P_2_ (9), and catalytically inactive members, which bind the active members, regulating their catalytic activity (10).

The group of SAC phosphatases contains 5 members with a Sac1 phosphatase domain, which dephosphorylates phosphoinositides (9, 11).Paladin contains two phosphatase signature motifs CXXGXGR, and seems to regulate insulin signaling, although no phosphatase activity has been detected (12).The group of INPP4 phosphatases contains 2 members, which modulate Akt signaling by dephosphorylating PI(3,4)P_2_ (9).The group of TMEM55 phosphatases contains 2 members, which generates PI5P by dephosphorylating PI(4,5)P_2_ at position 4 (13).

***Class II***

This class is composed of LMPTP (ACP1), which dephosphorylates pTyr residues (14) and SSU72, which dephosphorylates the synthetic substrate pNPP *in vitro* (15). The yeast ortholog SSU72 dephosphorylates pSer residues of RNA polymerase II (16, 17).

***Class III***

This class is composed of 3 cell division cycle-25 proteins (CDC25A, CDC25B, and CDC25C), which dephosphorylate cyclin-dependent kinases (CDKs) in pTyr and pThr residues, regulating the transition through cell-cycle steps (18).

**Asp-based**

This group contains 4 eyes absent phosphatases (EYA1, EYA2, EYA3, and EYA4), which use for the catalysis an Asp residue to dephosphorylate their substrates in pTyr residues (19–21). EYA phosphatases are transcriptional regulators during organogenesis (21, 22).

**His-based**

This group contains 2 UBASH3 and 3 acid phosphatases (ACPs), which dephosphorylates pTyr residues by the formation of a phospho-His intermediate during the catalysis (23–25).

Besides, cellular responses are also regulated by catalytically inactive PTPs (10, 26). The catalytic activity of PTPs is regulated by different mechanisms, including oxidation, oligomerization or phosphorylation and, interestingly, substrate accessibility is tightly regulated in space and time (27). Depending on the catalytic residue, PTPs can be classified in Cys-based (subdivided in Class I, II, and III), Asp-based and His-based (2) (Figure 1 and Box 1). Cys-based phosphatases represent the largest group, in which the catalytic Cys residue, inside the CX_5_R signature motif, performs a nucleophilic attack on the phosphate group of the substrate (28).

T cell activation is tightly controlled by a balance between phosphorylation and dephosphorylation. Available information on the mRNA expression in immune cells shows that peripheral blood T cells express around 70 genes coding for PTPs (19). Supporting an important coordinated role of PTPs during T cell immune responses, we have recently shown a regulated expression of a high percentage of PTPs during human CD4^+^ T cell polarization and Th1 effector function (29). Although PTPs are usually involved in down-modulating signaling in T cells, some phosphatases, such as CD45 or PTP-PEST, activate signaling molecules that promote T cell responses (30). It is important to note that there is still little information about the regulatory role of many PTPs in the signaling networks organized during T cell activation.

**Figure 2:**
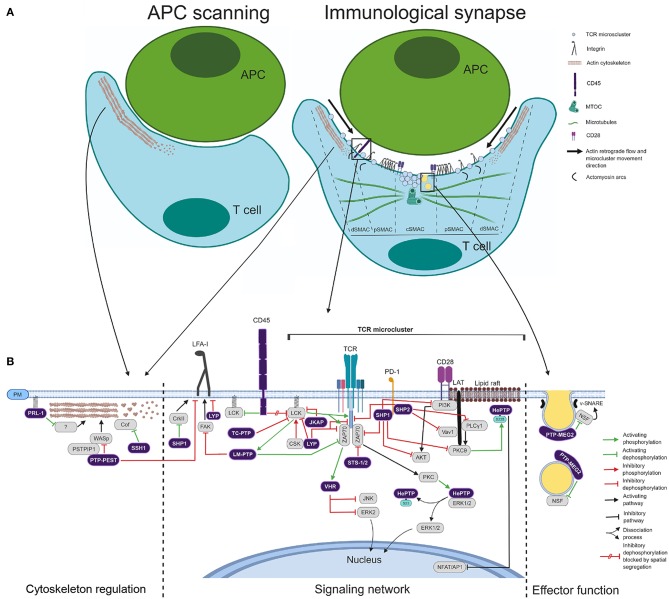
Coordinated action of PTPs during IS assembly. **(A)** Schematic of the APC T-cell scanning and IS assembly. The main molecules, cytoskeleton components, and endomembranes delivered at the IS are depicted. The cSMAC, pSMAC, and dSMAC are indicated. Right legend indicates different molecules and synapse elements. **(B)** Schematic of the regulatory role of PTPs in cytoskeleton, signaling network and effector function. Right legend indicates functional connectors used.

Activation of T cells in lymph nodes needs long-lasting contacts with antigen-presenting cells (APCs), in which antigenic peptides of pathogens presented in the context of the major histocompatibility complex (MHC) are engaged by the T cell receptor (TCR) (31). The initial engagement of the integrin LFA-1 and the TCR induces a stop signal, the spreading of the T cell over the APC and the formation of the immunological synapse (IS). The mature IS is characterized by a distal (d), a peripheral (p), and a central (c) supramolecular activation cluster (SMAC) (Figure 2A) (32–34). Engaged TCRs form microclusters at the periphery of the IS, where early signaling complexes are assembled (35). A retrograde flow of actin established at the dSMAC drives the movement of TCR microclusters toward the cSMAC, where the TCR is endocytosed for switching signals off (35–39). The pSMAC contains contractile actomyosin arcs, which also support centripetal movement of TCR microclusters, and a ring of LFA-1 for T cell-APC adhesion (34, 36, 40). It has been shown that distal actin flow is critical for sustaining activating signals during T cell activation (41, 42). The microtubule organizing center (MTOC) is also polarized to the IS within minutes after cell contact (Figure 2A). MTOC polarization and microtubule dynamics at the IS supplies both activating molecules from endosome-associated intracellular pools and secretory endosomes. This process sustains activating signals required for full T cell activation and ensures specific effector functions on the APC (43–46). Hence, PTPs regulating cytoskeleton and endosomal compartment dynamics are expected to be important regulators of T cell activation, IS assembly and effector functions. Nonetheless, there is still little information about the regulatory role of PTPs in cytoskeleton rearrangements and endosomal compartment dynamics at the IS. Besides, spatial and temporal regulation of PTPs and substrates at the IS is only barely known.

Antigenic stimulation in the context of the CD4^+^ T cell IS induces a clonal expansion and a cytokine-shaped differentiation process, which culminates in the formation of effector and memory cells. Effector cells exert their functions upon antigen re-stimulation in inflamed tissues. However, in addition to mediate adaptive immune responses against pathogens, CD4^+^ T cells also mediate autoimmune diseases (47). Many PTPs regulate T cell activation and are linked to autoimmunity (30, 48). Table 1 shows alterations in the gene sequence, expression levels, the function or the dynamics of PTPs that have been related to autoimmune diseases. In this review, we discuss the coordinated regulatory role of PTPs in signaling networks, the cytoskeleton and the endosomal compartment during IS assembly and effector functions of CD4^+^ T cells (Figure 2). We also discuss potential mechanisms mediating the involvement of these enzymes in autoimmune diseases.

**Table 1:** PTPs associated to autoimmunity.

**Phosphatase *Gene* (protein)**	**Function in T cell response**	**Related autoimmune disease (References)**	**Type of association**
*PTPRC* (CD45)	Regulation of TCR and cytokine signaling	MS, AH, RA (49–51)	SNPs associated to increased susceptibility
*PTPRN* (IA-2)	Not reported	T1D (52)	Acts as autoantigen
*PTPRN2* (IA-2β)	Not reported	T1D (53)	Acts as autoantigen
*PTPRT* (RPTPρ)	Not reported	SLE (54)	SNPs associated to increased susceptibility
*PTPN2* (TC-PTP)	Regulation of TCR and cytokine signaling	CD, RA, T1D (55–59)	SNPs associated to increased susceptibility
*PTPN6* (SHP1)	Regulation of TCR and cytokine signaling	PS (60)	Decreased expression in T cells of patients
		RA (61)	Altered dynamics to the IS
*PTPN11* (SHP2)	Regulation of TCR and cytokine signaling	UC (62)	SNPs associated to increased susceptibility
		SLE (63)	Increased activity in PBMCs of patients
*PTPN22* (LYP)	Regulation of TCR and LFA-I signaling	T1D, RA, SLE (55, 64–67)	SNPs associated to increased susceptibility
*DUSP1* (MKP-1)	Regulation of MAPK signaling	EAE (68)	Pathology diminished in KO mice
*DUSP5* (B23, hVH3)	Regulation of MAPK signaling	CIA (69)	Overexpression exerts therapeutic effect
*DUSP2* (PAC-1)	Regulation of MAPK signaling	UC (70)	Decreased expression in PBMCs of patients
*DUSP7* (MKP-X)	Not reported	RA (71)	Decreased expression in CD4 T cells of patients
*DUSP10* (MKP-5)	Regulation of MAPK signaling and T cell activation	CeD (72)	SNPs associated to increased susceptibility
*DUSP14* (MKP-6)	Regulation of TCR signaling	EAE (73)	Pathology enhanced in KO mice
*DUSP22* (VXH, JKAP)	Regulation of TCR signaling	SLE (74)	Decreased expression in T cells of patients
*DUSP23* (VHZ)	Not reported	SLE (75)	Increased expression in CD4 T cells of patients
*DUSP12* (HYVH1)	Not reported	MAS (76)	Mutations identified in patients
*PTEN* (PTEN)	Regulation of T cell activation	ALT, AHA, C (77)	Mutations identified in patients
*ACP1* (LMPTP)	Regulation of TCR signaling	CD, T1D, SLE (78–80)	Allelic variants associated to increased susceptibility
*SSU72* (SSU72)	Regulation of cytokine signaling	CIA (81)	Decreased expression in CD4 T cells of patients
*CDC25B* (CDC25B)	Not reported	RA (71)	Decreased expression in CD4 T cells of patients
*EYA4* (EYA4)	Not reported	RA (82)	SNP associated to improved response to treatment
*UBASH3A* (TULA)	Regulation of TCR signaling	T1D (83, 84)	SNPs associated to increased susceptibility

*AH, autoimmune hepatitis; AHA, autoimmune hemolytic anemia; ALT, autoimmune lymphocytic thyroiditis; C, colitis; CD, Crohn's disease; CeD, celiac disease; CIA, collagen-induced arthritis; EAE, experimental autoimmune encephalomyelitis; LN, lupus nephritis; MAS, multiple autoimmune syndrome; MS, multiple sclerosis; PS, psoriasis; RA, rheumatoid arthritis; SLE, systemic lupus erythematosus; T1D, type 1 diabetes; UC, ulcerative colitis; IBD, inflammatory bowel disease*.

## Regulation of IS-polarized Signaling and T Cell Differentiation

**SHP1** (***PTPN6***) is a negative regulator of TCR signaling. It dephosphorylates LCK and ZAP70 (85, 86), and its interaction with LCK assists in the discrimination between weak (self) and strong (pathogen-derived) ligands (87). It also inhibits co-stimulatory signals by dephosphorylating key enzymes such as PI3K, PKCθ, and AKT upon recruitment to checkpoints (88) (Figure 2B) and modulates Th polarization by limiting IL-4 signaling (89). In addition to negative regulatory roles during T cell activation, SHP1 increases T cell adhesion to the APC through activation of CRKII at the IS (Figure 2B) (90) and it is required for T cell development and generation of T cell repertoire (91).

Being a negative regulator of T cell activation, it is plausible that natural losses of SHP1 function could cause autoimmunity. In fact, the first example of autoimmunity caused by an alteration in a PTP was found in the motheaten mouse, in which a frameshift mutation generates SHP1 null mice (92). In humans, the expression of SHP1 is decreased in T cells of psoriatic patients when compared with skin T cells of healthy donors (60). In addition to this decreased expression, a delayed recruitment of SHP1 to the IS has been shown in CD4^+^ T cells of rheumatoid arthritis (RA) patients, a defect promoting T cell hyper-activation (61). It is tempting to speculate that ligand discrimination mediated by LCK binding (87) is not properly working in these patients. Thus, mutations, altered expression levels and delayed delivery to the IS of SHP1 have been linked to autoimmunity.

**SHP2** (***PTPN11***) also down-modulates T cell activation-associated processes at the IS. In particular, different publications demonstrate that SHP2 is associated to inhibitory receptors and checkpoints and controls T cell adhesion and activation (93–95). For example, it has been shown by total internal reflection fluorescence microscopy (TIRFM) that SHP2 is recruited to the Programed Death 1 (PD1) in TCR microclusters organized in cells interacting with antigen-presenting planar lipid bilayers containing PD1 ligands. PD1 ligation suppresses downstream signaling, including dephosphorylation of CD3ζ, VAV1, and pPLCγ1 (Figure 2B), and prevents CD28/PKCθ association. Thus, inhibition by PD1 seems to be mediated by confined SHP2 activity in competent TCR signaling sites (95). Consistent with this idea, the signaling lymphocytic activation molecule (SLAM)-adaptor protein (SAP) inhibits the checkpoint function by blocking pTyr residues targeted by SHP2 (96). However, the mouse model shows a non-essential role of SHP2 for the inhibitory function of PD1 in CD8^+^ T cells (97). Thus, alternative routes for PD1 inhibition should be further investigated. In addition to an inhibitory role, SHP2 seems to promote pre-TCR and TCR signaling in thymocytes (98, 99).

Two SNPs in the *PTPN11* gene have been related to increased susceptibility to ulcerative colitis (UC) in the Japanese population (62), but the phenotype of SHP2 remains to be determined. Considering that SHP2 is a negative regulator of TCR signaling, it is possible that these SNPs might decrease the expression or the catalytic activity of the phosphatase, or might perturb its proper delivery to TCR microclusters, resulting in enhanced T cell activation, which would lead to autoimmunity. Another report has nonetheless shown increased activity of SHP2 in peripheral blood mononuclear cells (PBMCs) of systemic lupus erythematosus (SLE) patients (63). Importantly, the authors show that pharmacological inhibition of SHP2 in T cells obtained from SLE patients decreases T cell proliferation and cytokine production and that treatment of lupus-prone mice with the inhibitor ameliorates the pathology. Whether SHP2 hyperactivity is a specific feature of SLE or takes place in more autoimmune diseases remains to be elucidated.

**CD45** (***PTPRC***) regulates the balance between the inactive closed state and the active open state of Src family kinases (SFKs). Precisely, CD45 dephosphorylates the inhibitory pTyr505 residue of LCK. This raises the so-called primed state, which can then generate the fully active open state by auto-phosphorylation of the pTyr394 residue located in the kinase domain. CD45 also dephosphorylates, although less efficiently, the pTyr394 residue, restraining the activation of the primed state (30, 100). Consistent with the requirement of sustained signaling by LCK during early T cell activation (101), CD45 is excluded from TCR microclusters (38) (Figure 2). In addition to this spatial segregation, contemplated by the “kinetic segregation” model of TCR triggering (102), expression levels of CD45 are critical for its regulatory role on LCK (103). At low levels, the inhibitory pTyr505 residue of LCK is more phosphorylated than the activating pTyr394 and, therefore, LCK is inhibited and TCR signaling reduced (103). Consistent with these findings, loss-of-function mutations of CD45 in humans cause severe combined immunodeficiency disease (104, 105). Several SNPs have also been associated to autoimmunity (49–51). How these SNPs affect the expression, the activity or the dynamic behavior of CD45 at the IS is currently unknown.

A role in the regulation of LCK activity has also been shown for **CD148** (***PTPRJ***) (106), which is upregulated upon T cell activation (29). Weiss' group has shown that overexpression of CD148 reduces TCR downstream PLCγ1 and LAT signaling (107). Substrate accessibility of this RPTP is blocked during IS assembly, being its function relevant during the detachment of T cells and APCs (108).

**LYP** (***PTPN22***), also known as **PEP** in mice, down-modulates early TCR signaling by dephosphorylating the activating pTyr residues of LCK (Y394) and ZAP70 (Y493), as well as CD3ζ (109) (Figure 2B). In agreement with these findings, PEP deficient mice show expansion and enhanced function of effector/memory T cells (110) and overexpression of LYP in Jurkat cells leads to decreased signaling and IL-2 transcription in response to TCR stimulation (111). Recently, Zamoyska's group have found a role of PEP in discriminating weak affinity self-peptides from strong agonists (112). This finding suggests that this phosphatase is important in maintaining tolerance and consequently in preventing autoimmunity.

Consistent with a role of LYP in preventing autoimmunity, a SNP in *PTPN22* human gene, which results in the LYP mutant R620W, confers increased risk to several autoimmune disorders, including RA, SLE, and Type 1 diabetes (T1D) (55, 64–67). Nonetheless, the molecular mechanism explaining this increased risk remains controversial. Some authors have shown that the LYP R620W variant is more effective in downregulating TCR signaling than the WT LYP (113, 114). These data suggest that the SNP might trigger autoimmunity by increasing the threshold of T cell activation, which might lead to survival of autoreactive T cells in the thymus, as shown for other mutations diminishing T cell signaling (115). Other authors, however, have shown that PEP interaction with CSK, an LCK-inhibitor, enhances LCK inactivation and, consequently, further inhibits downstream signaling (116, 117). Due to the fact that the R620W variant has a defective interaction with CSK (64), it is possible that the inability of the R620W variant to interact with CSK causes less effective TCR signaling inhibition. In addition, the function of LYP in T cells goes beyond regulation of TCR signaling. In humans and mice LYP/PEP seems to control T cell adhesion through LFA-1 (118–120) (Figure 2B). Remarkably, the R620W variant is not properly located to adhesion sites, acting as a loss-of-function mutant during LFA-1 regulation (120). Hence, cells carrying the SNP might have enhanced integrin-mediated signaling and adhesion, leading to altered T cell trafficking and activation.

We envisage that by studying the dynamic interaction between CSK and LYP (WT or R620W) during T cell activation will assist in our understanding about the regulatory role of LYP in health and disease. Consistent with this idea, T cell activation modulates the interaction of LYP and CSK (121), and it has been recently proposed that catalytic activity and spatial and temporal regulation might determine the function of LYP (122).

**PTP-PEST** (***PTPN12***) was initially proposed to be a negative regulator of T and B cell activation, adhesion and IS assembly by dephosphorylating and inhibiting different signaling, cytoskeleton and focal adhesion molecules (48, 123–125) (Figure 2B). However, Veillette's group has recently analyzed the conditional deficiency of PTP-PEST, discovering that this PTP is essential in secondary T cell responses probably by controlling the levels of phosphorylated PYK-2 and favoring cytokine communication through T cell homotypic interactions (126). Although there is no association of PTP-PEST with human autoimmunity, *Ptpn12* deficient mouse strains show less susceptibility to experimental autoimmune encephalomyelitis (EAE) (30).

Mustelin's group has shown that **HePTP** (***PTPN7***) negatively regulates MAP kinases ERK1 and ERK2 (127). HePTP directly binds inactive ERK in the cytoplasm and this inhibitory interaction is released by TCR-induced and PKA-catalyzed phosphorylation of HePTP at the Ser23 residue (128). Released ERK can be activated and enters the nucleus (Figure 2B), where it is dephosphorylated by the MKPs **PAC1** (***DUSP2***), **MKP-1** (***DUSP1***), and **MKP-2** (***DUSP4***), rendering the inactive form, which moves back to the cytoplasm and is bound by HePTP again (48). In addition to this phosphorylation of the Ser23 residue promoting the activation of ERK, the phosphorylation of the Ser225 residue by PKCθ recruits HePTP to lipid rafts at the IS and tempers transcription downstream TCR signaling (Figure 2B) (129). Thus, two pools of active HePTP downstream the TCR seems to have opposite regulatory roles for downstream signaling. Until now, there is no association of this PTP with autoimmunity or immunodeficiency.

**TC-PTP** (***PTPN2***) is a negative regulator of LCK (Figure 2B) and conditional *Ptpn2* knock-out in mouse peripheral T cells results in inflammation and autoimmunity. A pivotal role of this phosphatase, at least in mice, seems then to be the maintenance of T cell tolerance (130). Current knowledge indicates that homeostatic proliferation raises the pool of autoreactive T cells (131) and, interestingly, elevated expression of TC-PTP in naïve CD8^+^ T cells controls the threshold of the response to peripheral self-antigens and the homeostatic T cell proliferation. This might represent the mechanism by which TC-PTP prevents autoimmune diseases (132). TC-PTP and its closely related **PTP1B** (***PTPN1***) also regulate JAK-STAT signaling (133–136). Whether this regulation takes place in signaling complexes at the IS should be further investigated.

In humans, several SNPs in the *PTPN2* gene have been identified as susceptibility alleles for Crohn's disease (CD) (56, 57), arthritis (55, 58), and T1D (55, 59). The mechanism underlying the association of these SNPs with autoimmunity remains poorly understood. In this regard, CD4^+^ T cells from subjects carrying the autoimmunity-associated SNP rs1893217 show decreased expression of *PTPN2*, impaired signaling through the β chain of the IL-2 receptor, decreased phosphorylation of STAT5 and reduced expression of FOXP3 in response to IL-2 (137). Given that FOXP3 is the master regulator of Treg differentiation (138), the reduced expression of this transcription factor in response to IL-2 in cells carrying the SNP might increase the risk of autoimmunity by hampering Treg functions. It should be further investigated why reduced expression of TC-PTP causes these defects in signaling and in FOXP3 expression. Recently, some evidences suggest the development of autoimmunity due to a deficiency in TC-PTP in naïve and follicular helper CD4^+^ T cells, as well as in B cells (139).

Several members of the group of MKPs control intracellular signaling during T cell activation. **MKP-1** (***DUSP1***) is a positive regulator of JNK signaling and cell proliferation and activation. Consistent with this, deficient mice showed decreased T cell responses *in vivo* and *in vitro* and resistance to EAE (68). Conflicting results have been reported regarding the role of **DUSP5** in T cell responses. Some authors have shown that DUSP5 inhibits IL-2 dependent proliferation and function (140) and that its overexpression leads to the development of autoimmune symptoms (140). Nevertheless, a later study showed that overexpression of DUSP5 decreased Th17 responses, enhanced T regulatory phenotype and attenuated collagen-induced arthritis (CIA) in mice (69). Further investigation will be needed to clarify the role of DUSP5 in autoimmunity. **MKP-5** (***DUSP10***) also regulates T cell responses and autoimmunity in mice. T cells from MKP-5 deficient mice show lower proliferative capacity leading to resistance to EAE (141). MKPs can also be effectors of TCR triggering that control the downstream signaling network. For instance, phosphorylation by ZAP70 in response to TCR stimulation activates **VHR** (***DUSP3***), which dephosphorylates and inactivates ERK2 and JNK (142) (Figure 2B).

The MKPs **PAC1** (***DUSP2***) and **MKP-7** (***DUSP16***) regulate Th differentiation. PAC1 regulates STAT3 signaling and Th17 differentiation, and deficient mice show exacerbated pathology in a colitis model (70, 143). Consistent with the mouse model, expression of *DUSP2* is decreased in PBMCs obtained from UC patients, due to CpG methylation of the gene (70). Its role as an inhibitor of Th17 polarization suggests that this phosphatase might also be important in other autoimmune diseases, such as RA, in which Th17 responses are exacerbated (144). Expression of MKP-7 in naïve CD4^+^ T cells leads to enhanced expression of Th2 cytokines and transcription factors, while decreases Th1 differentiation (145). Whether this phosphatase is involved in the development of autoimmunity remains to be determined.

Two atypical DUSPs, **MKP-6** (***DUSP14***) and **JKAP** (***DUSP22***), are linked to T cell activation and autoimmunity. MKP-6 is a negative regulator of TCR signaling through dephosphorylation of TGF-β-activated kinase 1 (TAK1)-binding protein 1 (TAB1), and deficient mice show more susceptibility to EAE, due to enhanced cytokine production by T cells (73). JKAP inhibits LCK signaling by dephosphorylating the pTyr394 residue (Figure 2B) and deficient mice show enhanced T cell responses and greater susceptibility to EAE (146). In humans, the expression of JKAP is decreased in peripheral blood T cells of SLE patients, and this lower expression correlates with SLE disease activity (74). Giving an insight into the pathogenesis, JKAP-deficient T cells show enhanced production of complement components, and soluble ICAM-1 and VCAM-1 (74). Whether this function of JKAP is related to its role as a negative regulator of LCK remains to be determined.

Two class II PTPs, **LMPTP** (***ACP1***) and **SSU72** (***SSU72***), regulate intracellular signaling or differentiation. LMPTP is activated by LCK (147) and dephosphorylates the inhibitory pTyr292 residue of ZAP70 (Figure 2B) (148). This sustains ZAP70 signaling and reduces TCR degradation after endocytosis. LMPTP also controls the adhesion through LFA-1 by dephosphorylating the focal adhesion kinase (FAK) (Figure 2B) (149). No association of this phosphatase to autoimmunity has been reported. SSU72 overexpression reduces STAT3 signaling, Th17 differentiation, IL-17 production, and the incidence and severity of CIA, while attenuated expression of this phosphatase is found in CD4^+^ T cells of RA patients, likely due to hypermethylation of the gene (81). These findings suggest that increasing SSU72 levels or activity could be a therapeutic approach to control autoimmune disorders in which Th17 plays an important pathogenic role.

Two His-based PTPs, **TULA** (also known as **STS-2**, encoded by the gene ***UBASH3A***) and **TULA-2** (also known as **STS-1**, encoded by the gene ***UBASH3B***), have been shown to negatively regulate TCR/ZAP70 early signaling (Figure 2B). Interestingly, the double knock-out mice show increased susceptibility to EAE (24). These results suggest that TULA and TULA-2 control T cell activation threshold to avoid autoimmune responses. Consistent with this idea, SNPs in the *UBASH3A* gene have been linked to T1D in humans (83, 84). It should be addressed whether these SNPs result in decreased expression or activity, or in altered dynamics at the IS.

## Regulation of Cytoskeleton Dynamics by PTPs

During APC scanning, coordinated polymerization, depolymerization and severing of actin filaments generates an actin flow, which contributes mechanical forces for TCR triggering and LFA-1 activation (150, 151). There are two PTPs, **PTP-PEST** (***PTPN12***) and **PRL-1** (***PTP4A1***), that regulate actin polymerization during T cell activation (Figure 2). PTP-PEST dephosphorylates WASp (Wiskott-Aldrich Syndrome protein) through an interaction mediated by PSTPIP1 (Proline, Serine, Threonine Phosphatase Interacting Protein 1), controlling in this way actin polymerization and IS assembly (152). Recently, our group has proposed that PRL-1 regulates actin dynamics during the assembly of the IS (153). Upon APC encounter PRL-1 rapidly accumulates at scanning membranes where F-actin polymerizes. Treatment of T cells with procyanidine B3, a selective inhibitor of the catalytic activity of PRL-1 (154), reduces actin polymerization at the IS, suggesting the existence of PRL-1 substrates mediating this process. The substrate and the mechanism mediating the regulatory role of PRL-1 in actin dynamics at the IS deserve further research.

Slingshots (**SSHs**; SSH1, SSH2, and SSH3) dephosphorylate and activate cofilin (155), an actin severing and depolymerizing factor (156) (Figure 2). We have recently shown that SSH1 is delivered to the periphery of the IS in a similar way than cofilin (157, 158). Cofilin function is required to achieve a correct IS assembly and T cell activation (157). This suggests that SSH1 has an important role during T cell activation and this hypothesis should be further proved. Compared to naïve T cells, antigen-experienced (Ag-e) CD4^+^ T cells have higher levels of active cofilin, a less stiff cortical cytoskeleton and a stronger TCR signaling (158, 159). Higher actin dynamics in Ag-e cells might assist in the formation of large and mobile TCR clusters, in the serial TCR engagement of MHC molecules and in the release of molecular components to become part of signaling complexes (158). Consistent with the later idea, Toll-Like Receptor (TLR) signaling in B cells promotes cofilin/SSH1-dependent actin dynamics, reducing the spatial confinement of BCRs and improving the sensitivity and the efficiency of the response (160).

**MTMs** dephosphorylate PI3P and PI(3,5)P_2_, which regulate endocytosis, membrane trafficking and actin dynamics (161). Our group has found an upregulation of the **MTMR2** mRNA levels during the activation of T cells (29). Although the role of MTMR2 in immune cells has not been addressed, it has been reported that it interacts with Disc large-1 (DLG-1) (162), which controls NFAT activation, tubulin cytoskeleton dynamics and IS assembly by interacting with ezrin (163, 164). Thus, it is tempting to speculate that MTMR2 could also have a regulatory role in tubulin cytoskeleton dynamics and IS organization through interaction with DLG-1. Clearly, further research will be needed about the role of MTMR2 in the context of T cell activation.

## Regulation of Endosomal Compartment Dynamics by PTPs

Although the polarization of the endosomal compartment to the IS is essential for sustained T cell activation and effector function (43, 46), little is known about the role of most PTPs in this cell polarity. It is important to note that phosphorylation events during T cell activation might regulate the transport of intracellular pools of signaling molecules between membranes. For example, LCK is extracted from membranes (perhaps of the endosomal compartment) by binding the solubilizing factor UNC119, and phosphorylation of pTyr394 residue is critical for LCK release and delivery to the IS, a process mediated by the ciliary machinery ARL3/ARL13B (165). Thus, we predict a critical role of PTPs in the delivery of signaling molecules from the polarized endosomal compartment to the IS plasma membrane. This role might contribute to the required polarized segregation of signals during T cell activation.

A PTP clearly involved in the regulation of the endosomal compartment during T cell effector function is **PTP-MEG2** (***PTPN9***). Mustelin's group has shown that vesicle size and fusion to the plasma membrane are controlled by this classical NRPTP. PTP-MEG is activated by inositol phospholipids and, in its active form, dephosphorylates and activates the cytosolic protein NSF (N-ethylmaleimide-sensitive factor), which mediates vesicle fusion by disassembling cis complexes of SNAREs (soluble NSF attachment protein receptors) (166, 167). Constitutive dephosphorylation of NSF increases IL-2 secretion due to enhanced vesicle fusion; conversely, constitutive NSF phosphorylation reduces IL-2 production (166). Consistent with these data, knockout mice for *ptpn9* showed decreased TCR-dependent and independent IL-2, IFNγ, and IL-6 secretion by T cells due to a smaller number of secretory vesicles (167). It is clear that more research is needed about the role of other PTPs in endosomal compartment regulation and cytokine secretion. In this context, it is plausible to think that regulators of the metabolism of phosphoinositides, such as MTMs, will modulate endosomal dynamics in T cells. The consequence of perturbing the function of these enzymes should be explored.

## PTPs Related to Autoimmunity with Unknown Function in T Cells

Three classical RPTPs with no described regulatory role during T cell activation have been associated to autoimmunity. In humans, some SNPs of **RPTPρ** (***PTPRT***) have been found associated to SLE. However, the effect of these SNPs in the regulatory role of RPTPρ in different blood cell types has not been evaluated (54). The receptor type phosphatases **IA-2** (***PTPRN***) and **IA-2β** (***PTPRN2***) are autoantigens in T1D (52, 53). However, the mechanism for initiation of immune responses against beta cells in T1D should be further studied.

Altered expression levels of two DUSP and one class III PTP have been associated to autoimmunity. Increased levels of **VHZ** (***DUSP23***) have been found in peripheral blood CD4^+^ T cells of patients with SLE (75). These high expression levels correlate with increased expression of DNA methylation-related enzymes and with global CD4^+^ T cell DNA methylation. By contrast, we have recently reported that CD4^+^ T cells of RA patients have decreased mRNA levels of **MKP-X** (***DUSP7***) and **CDC25B** (71). Reduced MKP-X mRNA levels are restricted to patients with autoantibodies, while in the case of CDC25B, reduced mRNA levels are associated to the activity of the disease. It is not known whether reduced levels of MKP-X and CDC25B are a cause or a consequence of the pathology. The role of VHZ, MKP-X, and CDC25B in CD4^+^ T cells should be further investigated.

Finally, an allele of the gene coding for **EYA1**, an Asp-based PTP (Figure 1), has been shown to confer improved responses to RA treatment (82). In addition to the predictive value of this finding, it suggests an involvement of this PTP in immune responses during the treatment of patients.

## Perspective

CD4^+^ T cells are important orchestrators of immune responses and maintenance of tolerance. Hence, basic research on mechanisms regulating proper T cell activation should help us to understand the development and progression of autoimmune diseases. A substantial amount of studies has revealed that several PTPs expressed in CD4^+^ T cells regulate intracellular signaling and are related to autoimmunity, indicating a key regulatory role of these enzymes in immune responses in health and pathology. By contrast, little is known about the role of PTPs in regulating cytoskeleton rearrangements and endosomal dynamics triggered during T cell activation or effector function and further research will be clearly needed in this topic.

Molecular mechanisms mediating the regulation of T cell activation by PTPs remain to be fully determined. In particular, spatial and temporal regulation of PTPs during T cell activation is barely known. When and where are early signaling components targeted by PTPs? Are there mechanisms targeting PTPs to signaling microclusters like in the case of SHP2 to PD1? Other questions should be investigated. For example, how is the action of different functional pools of PTPs coordinated in downstream TCR signaling (such as HePTP, Figure 2B)? Or, what is the role of PTPs during the delivery of intracellular pools of signaling molecules to the IS? Advances in microscopy will enable us to precisely monitor the dynamic delivery of PTPs to the IS and the spatial and temporal regulation of PTPs and their substrates in health and disease. We predict that research about the dynamics of PTPs (including different autoimmunity-associated polymorphisms) during the organization of the IS will help us to fully understand the molecular mechanisms causing autoimmunity.

Finally, it is commonly unknown whether alterations in PTPs associated to autoimmunity (such as expression levels or dynamics) are a cause or a consequence of the pathology. In any case, particular autoimmune-related alterations might be used as efficient disease markers. Thus, research about PTPs in autoimmunity will pose basic knowledge to develop more effective therapies or new biomarkers for these pathologies.

## Author Contributions

PR-N conceived the review and wrote the final manuscript. PC-S, RR-M, OA-S, and SA-G prepare figures and wrote the first draft of the manuscript.

### Conflict of Interest Statement

The authors declare that the research was conducted in the absence of any commercial or financial relationships that could be construed as a potential conflict of interest.
